# Development and validation of Digi-MEE Instrument measuring online learning environments in medical education

**DOI:** 10.12669/pjms.39.6.8430

**Published:** 2023

**Authors:** Noor-i-Kiran Naeem, Siti Nurma Hanim Hadie, Irwan Mahazir Ismail, Muhamad Saiful Bahri Yusoff

**Affiliations:** 1 Noor-i-Kiran Naeem, FCPS, MSc. MEd. Department of Medical Education, ABWA Medical College, Pakistan. Department of Medical Education, School of Medical Sciences, Universiti Sains Malaysia, Malaysia; 2 Siti Nurma Hanim Hadie, Ph.D. Department of Anatomy, School of Medical Sciences, Universiti Sains Malaysia, Malaysia; 3 Irwan Mahazir Ismail, Ph.D. Centre for Instructional Technology & Multimedia, Universiti Sains Malaysia, Malaysia; 4 Muhamad Saiful Bahri Yusoff, Ph.D. Department of Medical Education, School of Medical Sciences, Universiti Sains Malaysia, Malaysia

**Keywords:** Online Learning Environment, Medical Education, Program Evaluation, Psychometrics, Validation

## Abstract

**Objectives:**

To develop and validate Digital Medical Education Environment (Digi-MEE) Instrument for measuring online learning environment in medical education.

**Methods:**

This series of studies involved 696 participants from May 2022 to December 2022. Following scoping review, invited modified e-Delphi experts developed consensus on the components and related items for measuring online learning environments. A panel of content experts and a group of medical students carried out content and response-process validation to determine Content Validity Index (CVI) and Face Validity Index (FVI) respectively. This was followed by exploratory and confirmatory factor analysis and reliability analysis to determine Digi-MEE’s factorial structure and internal consistency using SPSS version 26.0 and AMOS 26.0.

**Results:**

Delphi experts agreed upon nine components with 73 items of initial Digi-MEE version. CVI of Digi-MEE 2.0 was more than 0.90. with FVI of Digi-MEE 3.0 of 0.87. Exploratory factor analysis yielded 46 items with 57.18% variance. Confirmatory factor analysis led to the final Digi-MEE version containing 28 items within nine components with acceptable levels of goodness of fit indices. Overall Cronbach alpha of the final Digi-MEE was more than 0.90, and for the nine components ranged between 0.62 and 0.76.

**Conclusion:**

Digi-MEE is a promising valid and reliable instrument to evaluate online education environment in medical education. Content, response-process, factorial structure, and internal consistency evidence support the validity of Digi-MEE. Medical schools can use Digi-MEE as an evaluation tool for the continuous quality improvement of online learning environments.

## INTRODUCTION

A learning environment provides a setting for students to learn and engage with their teachers, staff, and peers, encompassing physical, social, and psychological aspects and reflects the curriculum being taught.^1^ The learning environment, also called the academic environment is crucial for providing quality education as it includes many factors that contribute to effective curriculum delivery, which is essential for training future professionals and student academic performance. Van Vendeloo et al demonstrated that the learning environment plays an indispensable role in promoting student well-being and preventing burnout among Belgian residents.^2^

The World Federation for Medical Education (WFME) has also recognized the learning environment as a pivotal element to evaluate in medical education programs.^3^ Several tools have been developed to gather students’ perceptions about their educational environments, such as the Dundee Ready Education Environment Measure (DREEM), Postgraduate Hospital Education Environment Measure (PHEEM) and John Hopkins Learning Environment Scale (JHLES).^4^-^6^ However, different educational contexts may require a different inventory that is tailored to the specific situation of the institution. Specific tools have been developed to measure the learning environment in a particular discipline, such as the Anatomy Education Environment Measurement Inventory (AEEMI)^7^ and the Healthcare Education Micro Learning Environment Measure (HEMLEM).^8^ Similarly, there is a need to design an inventory specifically for measuring the online learning environment in medical education.

Over the past twenty years, there have been some tools for measuring the quality of online learning environments in general education. However, these tools have limitations. For instance, the Constructivist Online Learning Environment Survey (COLLES) measures university students’ perceptions of various factors related to learning^9^ but lacks reliability and validity to be used further. The Technology-Rich Outcomes-Focused Learning Environment Inventory (TROFLEI) assesses different dimensions of high school classroom environments^10^, such as student cohesiveness, and computer usage, but is not comprehensive. Distance Education Learning Environment Survey (DELES)^11^ and Online Learning Environment Survey (OLLES)^12^ measure different domains of post-secondary and tertiary educational institutions’ environments, respectively. However, they do not focus on specific attitudes and skills that may be essential for effective online learning.

Recently, Mousavi A *et al* validated an E-learning educational atmosphere measure (EEAM) to measure online learning based on different factors, such as virtual education status, teaching skills, learner support, and professional ethics.^13^ However, the study involved a variety of students from various educational backgrounds. Syed emphasizes that it is necessary to develop an inventory that is highly contextualized and specifically targets medical students’ needs and requirements.^14^ Such an inventory will ensure a conducive online learning experience by measuring specific constructs according to what the millennials require for online education.

There is a need to develop consensus on key components of an effective online learning environment for undergraduate medical education, given the overlap in markers discussed above. Additionally, as digital technologies continue to advance, new concepts keep emerging that should be considered when measuring parameters such as computer literacy. Thus, it is necessary to take a systematic approach in developing and validating an instrument that can accurately measure the online learning environment including both synchronous and asynchronous learning environments. This study aims to develop and validate a new inventory named Digital Medical Education Environment (Digi-MEE) instrument to measure the online learning environment.

## METHODS

The study was conducted between May 2022 and December 2022, utilizing two main phases described by Yusoff et al.^15^ In the first phase, experts (i.e., medical educationists, medical teachers, and instructional designers) provided feedback on identified content and terminology related to the design of the instrument’s items and domains via modified e-Delphi study. In the second phase, the instrument was validated to establish its content validity, response process validity, construct validity, and internal consistency through serial cross-sectional surveys.

### Ethical Approval

**Fig.1:**
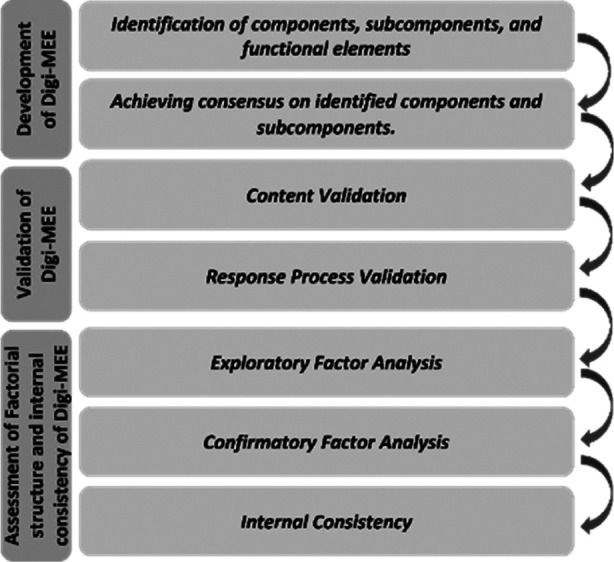
Flow chart for the development and validation of Digi-MEE.

The study received approvals from the Human Research Ethics Committee of Universiti Sains Malaysia (USM/JEPeM/21050350) on October 11, 2021, and November 3, 2022, and from the Review Boards of different data collection sites (Letter No. IEC/80-21, dated April 23, 2021, Letter No. MC/DME/609/2022, dated 2^nd^ September, 2022, Data Collection Approval dated September 16, 2022). Fig.1 presents the flow chart for the development and validation process of Digi-MEE.

### Development of Digi-MEE items

A scoping review was conducted leading to the generation of nine components and 25 sub-components of online learning environments in medical education.^16^ Based on the nine components; relevant items were agreed upon in a two-round modified e-Delphi study involving 18 invited experts from May 2022 to August 2022 using an online forum.

### Validation of Digi-MEE domains and items:

### Content Validation

The first version of Digi-MEE (Digi-MEE 1.0) was reviewed by a panel of 10 content experts (medical educationists from Canada, Malaysia, Pakistan, and Saudi Arabia with related experience in online learning environments for at least two years) from August 2022 to September 2022 after taking informed consent. Experts rated each item’s relevance to the measured component on a 4-point scale (1 = not relevant, 2 = somewhat relevant, 3 = quite relevant, 4 = highly relevant) and provided written comments on any items that required modifications or removal. The content validity index (CVI) was calculated based on item content validity index (I-CVI) and scale content validity index (S-CVI values). The acceptable I-CVI value has been set at a minimum of 0.78, while the acceptable S-CVI value should be at a minimum of 0.80.^17^ Based on the I-CVI values, items with I-CVI of 1.0 were accepted, items with I-CVI between 0.79 and 1 were re-discussed, and items with I-CVI less than 0.78 were rejected.^18^

### Response Process Validation

The second version of Digi-MEE (Digi-MEE 2.0) was reviewed by a panel of 21 medical students, as the potential users of the Inventory with at least one year of experience of online learning in a private medical school in Pakistan during September 2022. After explaining about the task and taking informed consent, the respondents rated each item’s clarity in the measured domain on a 4-point scale (1 = not clear, 2 = somewhat clear, 3 = quite clear, 4 = very clear) and gave written comments on any item requiring revision or removal. Face validity index (FVI) was calculated based on item face validity index (I-FVI) and scale face validity index (S-FVI values).

I-FVI is the proportion of instrument users (i.e., respondents) giving an item a clarity rating of 3 or 4, while S-FVI is the average of the I-FVI scores for all items on a scale. The acceptable I-FVI and S-FVI value has been set at a minimum of 0.78, and 0.80 respectively.^19^ Based on the I-FVI values, items with I-FVI of 1.0 were accepted, items with I-FVI between 0.79 and 1 were revised, and items with I-FVI less than 0.78 were rejected.

For the qualitative analysis, open comments given by the medical students related to each item were analyzed for any revision in sentence structure, format, spelling errors as well as any addition, deletion or shifting of any item to another component as suggested.

### Assessment of factorial structure and internal consistency

Digi-MEE 3.0 was administered to 230 undergraduate medical students in another private medical school in Pakistan via a cross-sectional survey from October 2022 to November 2022 after obtaining informed consent. Osborne and Costello indicated that sample size calculation for factorial analysis should be either in the ratio of 5:1 for the number of items or greater than 100.^20^ Participating students rated their experience of their school’s online learning platform using Digi-MEE 3.0 in a Likert scale from 1-4 (scale:1= strongly disagree ,2= disagree,3= agree, and 4= strongly disagree).

### Exploratory factor analysis (EFA)

EFA was performed through SPSS version 26.0 to determine the number of domains of the online learning environment and related items for Digi-MEE. Data suitability was determined by the Kaiser-Meyer-Olkin Measure of Sampling Adequacy (KMO) and Bartlett’s Test of Sphericity. KMO value > 0.7 indicates a good level of factor distinction based on an adequate sample, whereas significant Bartlett’s Test (p-value <0.05) demonstrates that the factor analysis is appropriate.^21^ The factors were extracted through the principal component axis (PCA) to check for total and cumulative variance for the nine extracted components and factor loading of each item. The initial component matrix was then rotated via Varimax with Kaiser normalizations after 28 iterations. For each component, Eigenvalues >1 and item factor loading values >0.40 signify convergent validity.^22^ Items with factor loading <0.4 were removed. The items were shifted in respective components where they loaded maximally, after being reviewed by two independent researchers (NKN and MSBY) who considered the theoretical meaning of each component. This led to the development of Digi-MEE version 4.0.

### Confirmatory factor analysis (CFA)

CFA was carried out on the data obtained from a cross-sectional survey of 450 undergraduate medical students from a public sector university in Pakistan for their ratings of their medical school’s online learning environment using Digi-MEE 4.0 from November 2022 to December 2022. The sample size was determined based on the criteria of 10 participants per item for an adequate sample for CFA.^23^

Data was analyzed using SPSS version 26.0 and Analysis of Moment Structure (AMOS) version 26.0. Model Chi-square goodness of fit (set at 0.05) demonstrated model fit. Approximate fit indexes were applied to check the available data for measurement model fitness. For absolute fit indexes, Goodness of Fit Index (GFI) (model fit >0.9) and root mean square error of approximation (RMSEA) (model fit=RMSEA<0.08 and Root mean squared residual-RMR<0.05) were used. For incremental fit measurement, Comparative fit index (CFI) (model fit >0.9), Normed fit index (NFI) (model fit >0.9), and incremental fit index (IFI) (model fit >0.9), and Tucker Lewis fit index (TFI) (model fit >0.9) were used.^23^ Parsimonious fit was measured through Chi-Square/Degree of Freedom (Chi Sq/df).^13^

### Internal Consistency

Convergent Validity was determined with the size of factor loading (0.5 or more), as well as composite reliability (CR). Composite Reliability (CR) was calculated on Microsoft Excel using formulae given by Fornell and Larckers.^24^ Values of CR of more than 0.6 indicate good construct reliability and adequate Convergent Validity.^25^

Following CFA, data was used to perform reliability statistics to assess the internal consistency of final version of Digi-MEE (Digi-MEE 5.0). Cronbach’s alpha coefficient was determined as the measured parameter to determine internal consistency. Cronbach’s alpha values between 0.7 and 0.9 are considered to represent high internal consistency and values between 0.6 and 0.7 are considered satisfactory.^15^

## RESULTS

This study involved a total of 696 participants, including 25 experts from eight different countries (Canada, Egypt, Iran, Malaysia, Pakistan, Saudi Arabia, Kuwait, and the USA) and 671 undergraduate medical students from four different institutions in Pakistan. Table-I shows demographic data of participants involved in different phases of the study.

**Table-I:** Demographic details of the participants involved in different phases of study.

Phase 1: Design phase			Phase 2: Validation phase		

Demographic data	Modified Delphi Rounds	Content Validation	Response Process Validation	Exploratory Factor Analysis	Confirmatory Factor Analysis
Total number of participants (n)	15	10	21	200	450
Participants	Medical educationists, instructional designers, Information Technology experts	Medical Educationists	Medical Students	Medical Students	Medical Students
Institutional Affiliation (Public/private)	Both	Both	Private	Private	Public
Location	Canada, Eygpt, Iran, Malaysia, Pakistan, Saudi Arabia, Kuwait, USA	Canada, Malaysia, Pakistan, Saudi Arabia	Pakistan	Pakistan	Pakistan
Designation	3	2	-	-	-
Professor	4	2	-	-	-
Assoc. Prof.	2	3	-	-	-
Assist. Prof.	2	3	-	-	-
Lecturer	2	-	-	-	-
Director	2	-	-	-	-
Consultant	-	-	21	200	450
Medical Student	-				
Age(years)					
<25	-	-	3	200	11
25-34	3	1	18	-	439
35-44	4	4	-	-	-
45-54	4	3	-	-	-
55-64	4	2	-	-	-
>65	-	-	-	-	-
Gender					
Male	9	4	10	94	250
Female	6	6	11	106	200

### Development of Digi-MEE items:

### Identification of components, subcomponents, and functional elements

Literature review led to the identification of nine components and 25 subcomponents with 73 functional elements for effective online learning environments in medical education.^14^ Nine components included content curation, cognitive enhancement, digital capability, learning facilitator, learner characteristics, institutional support, pedagogical practices, technological usability, and social representations.

### Achieving consensus on identified components and subcomponents

Fifteen out of 18 invited experts participated in the modified e-Delphi and the data about their consensus is presented in Table-III. The names of three main components were revised after consultation (Pedagogical Practices to be changed to Cybergogical Practices, Technological Usability to be changed to Platform Usability and Learning Facilitator to be changed to Facilitation Dynamics). Definitions of three main components (Institutional Support, Cognitive Enhancement and Content Curation) and two subcomponents (Content Selection and Content Organization) were revised and presented in round two.

**Fig.2:**
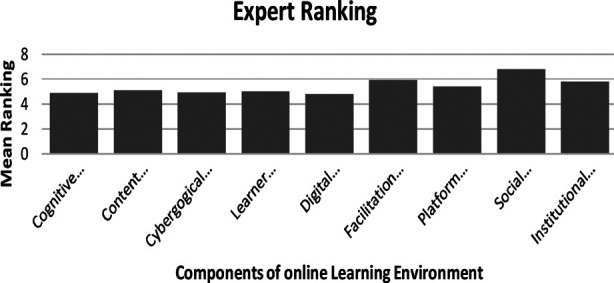
Expert ranking for the components of online learning environments in medical education.

Expert ranking of nine components in Round Two is shown in Fig-2. The component of ‘Social representations’ was ranked the highest influencing online learning environments while ‘Digital capability’ was ranked the lowest.

### Validation of Digi-MEE:

### Content Validation

Out of 73 items, five items having I-CVI of less than 0.78 were removed and 21 items were removed due to similar meaning. Five items were shifted to a different component upon expert suggestions while 17 items were reworded for grammatical inadequacies and clarity as suggested by experts (Table-II). Digi-MEE 2.0 thus had 47 items. Overall, the S-CVI/Ave by score improved from 0.95 to 0.98 in Digi-MeE 2.0, S-CVI/Ave by proportion ranking by experts from 0.95 to 0.98 and S-CVI/UA from 0.69 to 0.79.

**Table-II:** Modifications in Digi-MEE questionnaire in different phases of the study.

	Modified Delphi Study	Content Validation Study	Response Process Validation Study	Exploratory Factor Analysis	Confirmatory Factor Analysis
Digi-MEE Version	0	1.0	2.0	3.0	4.0
Total components	9	9	9	9	9
Total items in beginning	-	73	47	46	46
Items accepted without change	-	25	29	0	-
Items accepted after modification	-	17	16	0	-
Items removed	-	26	2	0	18
New items added	-	5 shifted to another domain	1	17 shifted to another domain	-
Final Items	73	47	46	46	28

**Table-III:** Agreement levels on component definition and appropriateness in Modified Delphi Study.

	Round 1	Round 2
***Agreement Level on definition***		
86.67%	Cognitive Enhancement, Pedagogical Practices	Institutional Support
93.33%	Content Curation, Digital Capability, Technological Usability, Learning Facilitator, Institutional Support	Cognitive Enhancement
100%	Learner Characteristics, Social Representations	Content Curation
***Agreement level in appropriateness and applicability to Practice***		
93.33%	Content Curation,	-
100%	Cognitive Enhancement, Pedagogical Practices, Learner Characteristics, Digital Capability, Technological Usability, Learning Facilitation, Social Representations, Institutional Support	-

### Response Process Validation

Out of 47 items checked for content clarity and comprehensibility, two items having I-FVI of less than 0.78 were removed while one item was added as suggested. Study participants indicated revisions in grammatical structure in 16 items. Digi-MEE 3.0 had 46 items. The S-FVI/Ave by score of Digi-MeE was 0.87, and S-FVI/Ave by proportion ranking by participants was 0.83.

### Assessment of factorial structure and internal consistency:

### Exploratory factor analysis

The Kaiser-Meyer-Olkin Measure of Sampling Adequacy (KMO) index was 0.861 which demonstrated the appropriateness of data. Bartlett’s Test of Sphericity was <0.001 indicating adequacy of data. Communalities were measured after PCA with an average communality of 0.572 which is a reasonably acceptable value. PCA demonstrated variance of 57.18% achieved by nine components. All variables loaded on their respective factors with values of >0.40 threshold. All the components had Eigenvalues>1 and Cronbach’s alpha values ranging from 0.620 to 0.794 (Table-IV).

**Table-IV:** Range of Factor Loadings, Eigenvalues and Cronbach’s alpha of components of Digi-MEE 3.0.

Sr. No.	Component	Range of Factor Loading	Eigenvalue	Cronbach’s alpha
1	Cognitive Enhancement	0.427-0.601	3.612	0.748
2	Content Curation	0.461-0.709	3.033	0.794
3	Cybergogical Practices	0.402-0.661	2.972	0.729
4	Digital Capability	0.487-0.687	2.902	0.762
5	Social Representation	0.429-0.625	2.878	0.620
6	Platform Usability	0.440-0.703	2.849	0.631
7	Institutional Support	0.557-0.721	2.706	0.712
8	Facilitation Dynamics	0.472-0.655	2.674	0.722
9	Learner Characteristics	0.414-0.634	2.672	0.773

### Confirmatory factor analysis

**Fig.3:**
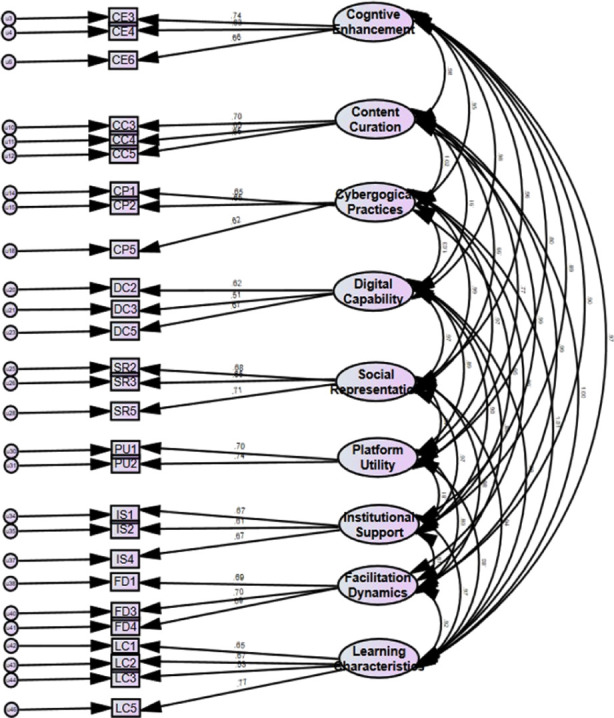
Standardized Factor Loadings of Digi-MEE Constructs based on the final model.

Indicator variables (items) loaded significantly on their respective components are shown in Fig-3. Minimum loading was observed by item LC2 on Learning Characteristics (0.508). Seven items had loadings between 0.5 to 0.59. Twenty-seven items had loadings between 0.6 to 0.69. Seven items out of 46 had loadings >0.69. The maximum loading on the factor was observed by item LC4 on Learning Characteristics (0.749). There was high correlation and low covariances between the latent factors in the final model (Fig.3).

Initial model fit indices for the Model-1 of Digi-MEE (v.5.0) revealed RMSEA value of 0.067 and GFI of 0.801(Table-V). The Digi-MeE model indices were analyzed to identify any variables with high covariances with other variables in the same latent factors. Eleven such variables were identified (CE1, CE2.CE5, CC2, CP3, DC4, SR4, PU3, PU4, IS3, and LC4) and removed to run the goodness of fit test in obtained Digi-Me Model 2(v.5.1). This led to the RMSEA value of 0.061 and GFI of 0.858. Next variables with covariances > 0.4 with other variables were identified. Seven variables were identified and removed (DC1, CC1, CC6, LC4, FD2, SR1 and CP4). The final Digi-MEE model (v.5.2) demonstrated GFI of 0.902 and RMSEA of 0.057. The final number of items in Digi-MEE with good model fitness was 28.

**Table-V:** Results of confirmatory factor analysis for Digi-MEE for model fit.

Model	Acceptable Values	GFI	RMSEA	RMR	CFI	NFI	IFI	TLI	Chi Sq/df	p-value

		>0.9	<0.08	<0.05	>0.9	>0.9	>0.9	>0.9	<5	<0.05
Model 1 v.5.0	46 items	0.821	0.065	0.035	0.851	0.791	0.852	0.836	3.013	< 0.001
Model 2 v.5.1	35 items	0.858	0.061	0.032	0.885	0.83	0.886	0.87	2.676	< 0.001
Model 3 v.5.2	28 items	0.902	0.057	0.03	0.926	0.883	0.927	0.91	2.467	< 0.001

### Internal Consistency

Overall Cronbach’s alpha of Digi-MeE instrument was 0.952 which depicts excellent reliability. The composite reliability of the Digi-MeE instrument was calculated to be 0.7 value for internal consistency (Table-VI).

**Table-VI:** Reliability analysis of the 28 items of the Digi-MEE based on the final model.

Item	^B^Domain	^A^Corrected Item-total correlation	^A^Cronbach’s Alpha if item deleted	^A^Cronbach’s Alpha	^C^Composite Reliability-CR
SR2	Social Representation	0.570	0.605	0.724	0.683
SR3		0.538	0.643		
SR5		0.525	0.659		
CC3	Content Curation	0.555	0.567	0.703	0.705
CC4		0.516	0.618		
CC5		0.490	0.649		
CE3	Cognitive Enhancement	0.577	0.686	0.756	0.716
CE4		0.542	0.706		
CE6		0.572	0.690		
CE7		0.523	0.716		
CP1	Cybergogical Practices	0.484	0.567	0.671	0.672
CP2		0.524	0.517		
CP5		0.436	0.636		
DC2	Digital Capability	0.432	0.525	0.625	0.632
DC3		0.382	0.596		
DC5		0.486	0.448		
PU1	Platform Utility	0.523	-	0.68	0.687
PU2		0.523	-		
IS1	Institutional Support	0.499	0.592	0.686	0.687
IS2		0.482	0.613		
IS4		0.522	0.564		
FD1	Facilitation Dynamics	0.571	0.638	0.737	0.737
FD3		0.567	0.643		
FD4		0.543	0.671		
LC1	Learning Characteristics	0.580	0.674	0.751	0.754
LC2		0.550	0.691		
LC3		0.448	0.745		
LC5		0.609	0.657
Overall Cronbach’s alpha for 28 items				0.952	
Composite Reliability				0.697	

A Reliability analysis: Cronbach’s Alpha Coefficient,

B Domains were determined based on basis of expert validation and exploratory factor analysis DCR (Composite Reliability) was calculated based on formula given by Fornell & David^22^

## DISCUSSION

 This study provides four essential pieces of evidence to support the psychometric credential of Digi-MEE to measure the digital learning environment in medical education: content validity, response process validity, internal structure validity and internal consistency. The final validated 28-item Digi-MEE instrument with Likert scale is shown in Table-VII.

**Table-VII:** Final Validated 28-item Digi-MEE Instrument with Likert Scale.

28-Item Digi-MEE Instrument					

Q.No.	Item Statement	Strongly Disagree	Disagree	Agree	Strongly Agree
1	I feel this online platform is relevant to my learning needs.				
2	The online platform provides opportunities to promote my independent learning.				
3	The content is presented appropriately to enhance my understanding.				
4	I understand the orientation given before the task easily.				
5	The online learning platform provides clear learning outcomes for the given course I am enrolled in.				
6	My online activities with others are monitored in this online learning platform.				
7	The online platform allows me to exchange information with my peers/facilitators easily.				
8	The activities on the online platform allow me to interact with others.				
9	This online platform provides suitable assessment methods to facilitate my learning.				
10	I can communicate and collaborate with my peers/facilitators on this online platform easily.				
11	I can see my basic profile information as well as that of my peers/facilitators on this online platform.				
12	I can manage my digital screen time on an online learning platform.				
13	I feel I am part of the online learning community.				
14	I am provided with timely feedback on my work.				
15	I give feedback about courses which I am enrolled in online learning platforms.				
16	The online platform encourages me to participate in online learning activities in a professional and ethical manner.				
17	The online content can be accessed with ease.				
18	The platform interface is simple and follows a consistent design.				
19	The online platform usage policies are widely disseminated among students.				
20	The online platform rules and regulations are informed to me.				
21	The institution provides training to me for using online platforms appropriately.				
22	The online content is organized in an engaging manner.				
23	The facilitator(s) selects the appropriate tool for teaching us online.				
24	The facilitator(s) provides positive encouragement to me during classes.				
25	I show interest in learning about a given topic in an online learning platform.				
26	My learning is supported by this online learning platform.				
27	I try my best to put in effort during online activities.				
28	The online platform for learning is well accepted by me.				

Firstly, Digi-MEE instrument demonstrated a high level of content validity as the content was developed through a robust and systematic process based on the evidence and expert consultation.^26^ The nine components of Digi-MEE are infact reflection of our TELEMED (Technology Enhanced Learning Environments in Medical Education) model.^16^ These components, namely cognitive enhancement (four items), content curation (three items), cybergogical practices (three items), learner characteristics (four items), digital capability (three items), platform usability (two items), facilitation dynamics (three items), social representation (three items), and institutional support (three items) are well aligned with the issues on online learning environment addressed in the current literature. For example, digital capability represents the much-needed requirement for the medical student to not only develop digital skills but also demonstrate self-management in online environments.^27^ In contrast, previously available online learning environment inventory focused on development of computer competence only.^9^,^10^

Previous inventories for digital atmosphere measure social interaction as an essential component.^9^,^10^ As an expansion to this platform, the ‘social representations’ domain of Digi-MEE instrument addresses the need to demonstrate ‘ethical and professional practices while interacting in online platforms’ for a safe and conducive learning environment.^26^ Ensuring that the students demonstrate ethical and professional practices in online learning environments can lead to professional digital identity formation of medical students.^28^

The Digi-MEE instrument presents ‘facilitation dynamics’ as a set of activities to be ensured by the facilitator in online learning environments that can support learners in their education. Instead of focusing on the facilitator support and teaching skills as mentioned in previous inventories^9^,^12^, Digi-MEE provides a guideline of validated items for the facilitator to ensure while managing student-centered learning. Similarly, the platform usability and institutional support, vaguely mentioned before^13^, are now clearly presented with items to support students’ learning in digital platforms. It has been proven that well designed e-learning platforms influence student satisfaction and improve performance while continuous institutional support can guide and train both learners and facilitators to use these online platforms up to their potential in an effective manner. The continuous integration of online learning in medical schools also necessitates the development of robust institutional policies for online learning and their dissemination to the users of online learning platform highlighted in Digi-MEE instrument.^29^

The next three components are interlinked and emphasize upon the need to have curated content leading to cognitive enhancement via effective cybergogical practices in online platforms. The content remains one of the important interactions with the students when learning online.

However, with introduction of open educational resources, content is here by proposed to be ‘curated’ instead of being made from scratch as discussed by the Experts in Content Validity phase.^30^ The curated content should engage the learner in online platforms to provide meaningful learning and should be organized in a manner for easy comprehension.^31^ Using this curated content, facilitators can provide activities which allow learners to interact and collaborate with each other to carry out their learning and assessment in online learning platforms. Lastly, ‘learner characteristics’ of Digi-MEE evaluates students’ perception of interest, effort and motivation for online learning which have also been previously used as parameters in other instruments.^32^ Hence, Digi-MEE presents constructive alignment of the prevailing needs of online learning environments within its nine main components, indicating adequate and relevant coverage of required content for online learning environments in medical education.

Secondly, the response process validity of Digi-MEE was high as the users easily understood it. This is an important finding as this study utilized students as participants who are end users of the finalized Digi-MEE instrument and their input is necessary for questionnaire development at this stage. In addition, the open comments allowed for any needed item revisions and/or removal as suggested by the participants.^19^

Thirdly, Digi-MEE demonstrates a multi-dimensional factor and reached a good model fit as all goodness of fit indices met the acceptable level. EFA of Digi-MEE instrument demonstrated sample adequacy (KMO=0.861, Bartlett’s test of sphericity=0.000) with acceptable factor loadings of all items in extracted factors (>0.40).^21^ Literature debates of varying threshold levels for item factor loadings in EFA depending on the sample size of collected data. Hair et al. proposed a loading threshold of 0.4 for sample size of 200.^33^

We had invited 230 participants in our study out of which 200 responded to fill the survey. Therefore, a loading threshold of 0.4 applies to our study. Yong & Pearce explain that a high threshold level of 0.5 or more ensures that only items with strong factor loadings are considered to load onto a factor, making it easier to interpret the factor structure and the meaning of each factor.^22^ However, high threshold may disregard subtle relationships between the variables which we did not want to miss. Having lower thresholds helped us reduce the losing information about the underlying dimensions in the data.^33^

CFA of Digi-MEE depicts minimum discrepancy per degree of freedom (CMIN/DF) ratio of 2.46(<3.00) which represents a satisfactory fit which is close to value of 2.63 in study performed by Butt et al.^34^ Further in the same study, the goodness of fit index was 0.911 which is comparable to our model’s good for fitness index (GFI). A GFI >0.95 indicates a good model fit, hence our model has an acceptable fit. Another important component in covariance structure modeling is the RMSEA with acceptable values being less than 0.05. In our study, the RMSEA was 0.057 and RMR WAS 0.03 representing constructs one-dimensionality.

Finally, the internal consistency of Digi-MEE was acceptable as the Cronbach alpha and composite reliability values were more than 0.6. Our validated questionnaire demonstrated overall Cronbach’s alpha of 0.965, showing excellent internal consistency of Digi-MEE instrument as per criteria defined by George and Mallery’s rule of thumb.^35^ The Cronbach’s alpha of the subscale ranged from 0.623 to 0.751, again ranging from acceptable values to relatively high internal consistency (2). When compared with alpha values demonstrated by Rahayu, *et al*. validation study for Online Classroom Learning Environment Inventory (OCLEI)^36^, our subscale of “social representation” had similar reliability as their scale’s “interaction” subscale (α =0.724). However, the platform utility of our scale (*α* =0.685) was slightly less than what the above study had measured (*α* =0.744). One of the items out of the two in this subscale relates to access to the platform while the second item relates to platform interface. On the other hand, our subscale of ‘Facilitation Dynamics’ (α =0.737) is more reliable as compared to OCLEI’s subscale of lecturer support (*α* =0.718).

Major strengths of the study include recommended best practices and guidelines for development and validation of Digi-MEE instrument. The nine domains on online learning environment in medical education were derived from multisource evidence including theory, literature, input from experts and learners. The experts involved in the modified e-Delphi and content validation study were from diverse geographical backgrounds as well as professional backgrounds involved in online education. This diversity of experts makes Digi-MEE a suitable instrument for different institutions all over the world.

### Limitations

It includes study sample for factor analysis and reliability analysis from one country only. Future validation studies should be done in different countries. Also, the sample was confined to undergraduate medical students. Further validation must be done before Digi-MEE is used for postgraduate medical students.

## CONCLUSION

This study introduced a new measurement tool, namely Digi-MEE, to evaluate the digital learning environment in medical education. The validity of Digi-MEE is demonstrated through its content, response process, factorial structure, and internal consistency. This tool can be used by medical schools to evaluate their digital learning environment for continuous quality improvement. Nevertheless, more validation research should be conducted to verify the validity of Digi-MEE in different educational settings.

### Abbreviations:

**AMOS:**Analysis of Moment Structure.

**CFA:**Confirmatory Factor Analysis.

**CVI:**Content validity index.

**Digi-MEE:**Digital Medical Education Environment.

**EFA:**Exploratory Factor Analysis.

**FVI:**Face validity index.

**KMO:**Kaiser-Meyer-Olkin Measure of Sampling Adequacy.

**SPSS:**Statistical Package for Social Sciences.

### Authors’ Contribution:

**NKN:** Conceived the study and initiated the writing of the paper. Generated the TELEMED Framework for Digi-MEE and configured their relationship with the findings of the scoping review. Did data collection and analysis from the different study sites.

**NKN, MSBY, IM** and **SNH:** Performed the scoping review, selected the relevant papers, compiled them in tables, modified the e-Delphi study, and laid out the Digi-MEE in its final form after serial analysis.

**MSBY, SNH** and **IM:** Reviewed the framework carefully.

**NKN, MSBY, SNH** and **IM:** Reviewed the data analysis and were consulted for thematic analysis for qualitative data where required.

All authors read and approved the final manuscript.
